# Taking Advantage of the Selectivity of Histone Deacetylases and Phosphodiesterase Inhibitors to Design Better Therapeutic Strategies to Treat Alzheimer’s Disease

**DOI:** 10.3389/fnagi.2019.00149

**Published:** 2019-06-21

**Authors:** Mar Cuadrado-Tejedor, Marta Pérez-González, Cristina García-Muñoz, Damián Muruzabal, Carolina García-Barroso, Obdulia Rabal, Víctor Segura, Juan A. Sánchez-Arias, Julen Oyarzabal, Ana Garcia-Osta

**Affiliations:** ^1^Neurobiology of Alzheimer’s Disease, Neurosciences Program, Center for Applied Medical Research (CIMA), University of Navarra, Pamplona, Spain; ^2^Department of Pathology, Anatomy and Physiology, School of Medicine, University of Navarra, Pamplona, Spain; ^3^Health Research Institute of Navarra (IDISNA), Pamplona, Spain; ^4^Small Molecule Discovery Platform, Molecular Therapeutics Program, Center for Applied Medical Research (CIMA), University of Navarra, Pamplona, Spain; ^5^Bioinformatics Unit, Center for Applied Medical Research (CIMA), University of Navarra, Pamplona, Spain

**Keywords:** Alzheimer’s disease, multitarget therapy, histone deacetylase, phosphodiesterase, memory

## Abstract

The discouraging results with therapies for Alzheimer’s disease (AD) in clinical trials, highlights the urgent need to adopt new approaches. Like other complex diseases, it is becoming clear that AD therapies should focus on the simultaneous modulation of several targets implicated in the disease. Recently, using reference compounds and the first-in class CM-414, we demonstrated that the simultaneous inhibition of histone deacetylases [class I histone deacetylases (HDACs) and HDAC6] and phosphodiesterase 5 (PDE5) has a synergistic therapeutic effect in AD models. To identify the best inhibitory balance of HDAC isoforms and PDEs that provides a safe and efficient therapy to combat AD, we tested the compound CM-695 in the Tg2576 mouse model of this disease. CM-695 selectively inhibits HDAC6 over class I HDAC isoforms, which largely overcomes the toxicity associated with HDAC class 1 inhibition. Furthermore, CM-695 inhibits PDE9, which is expressed strongly in the brain and has been proposed as a therapeutic target for AD. Chronic treatment of aged Tg2576 mice with CM-695 ameliorates memory impairment and diminishes brain Aβ, although its therapeutic effect was no longer apparent 4 weeks after the treatment was interrupted. An increase in the presence of 78-KDa glucose regulated protein (GRP78) and heat shock protein 70 (Hsp70) chaperones may underlie the therapeutic effect of CM-695. In summary, chronic treatment with CM-695 appears to reverse the AD phenotype in a safe and effective manner. Taking into account that AD is a multifactorial disorder, the multimodal action of these compounds and the different events they affect may open new avenues to combat AD.

## Introduction

Alzheimer’s disease (AD) is a multifactorial neurodegenerative disorder for which no effective treatment has yet been found, despite the effort and resources invested to date. One reason for this failure may be that most of the efforts to develop therapies have been directed towards single pathways and mainly, the amyloid pathology. However, the complex nature of this disease means that multiple events are likely to be implicated in its progression and thus, effective therapies must modulate several targets, as is now being considered with state-of-the-art treatments for many cancers and AIDS. Moreover, the focus in AD is now shifting from the pathways that directly decrease the amyloid pathology to address those that dampen the tau pathology or that effect synaptogenesis (Cummings et al., 2018).

We recently validated the efficacy of a novel multitarget therapy for AD that focused on the concomitant inhibition of histone deacetylases (HDACs) and a phosphodiesterase 5 (PDE5). The approach was validated using reference compounds (tadalafil and vorinostat), and with a new drug and novel chemical entity (NCE), CM-414, which displays moderate class I HDAC inhibition, and more potent HDAC6 and PDE5 inhibition (Cuadrado-Tejedor et al., 2015, 2017; Rabal et al., 2016). We demonstrated that chronic treatment of Tg2576 mice (a well-studied model of AD) with CM-414 diminished the accumulation Aβ and pTau in the brain, reversing the decrease in dendritic spine density on hippocampal neurons and the cognitive deficits in these mice. These effects were at least in part produced by inducing the expression of genes related to synaptic transmission. Interestingly, we demonstrated that these therapeutic effects persisted 1 month after the completion of a 4-week treatment period (Cuadrado-Tejedor et al., 2017).

CM-414 was designed taking into account that class I HDACs and HDAC6 (class IIb) are the HDACs most likely to be involved in AD-memory related dysfunction (Ding et al., 2008; Guan et al., 2009; Mahady et al., 2019). The inhibition of class I HDACs, and particularly that of HDAC2, seems to be essential to restore memory by remodeling chromatin and enhancing gene expression (Guan et al., 2009; Singh and Thakur, 2018). However, the inhibition of HDAC class I isoforms has also been associated with cytotoxicity (Subramanian et al., 2010), precluding their chronic use. Recently, a new chemical series of HDAC1 and 2 inhibitors designed to inhibit the HDAC-CoREST co-repressor complex were seen to have lower toxicity, while maintaining the beneficial effects in terms of synaptic plasticity (Fuller et al., 2019). By contrast, the inhibition (or reduction) of HDAC6, a cytoplasmic HDAC isoform that regulates microtubule behavior and stability *via* α-tubulin acetylation (Hubbert et al., 2002), seems to promote tau and Aβ clearance, thereby ameliorating the memory deficits in AD models (Cook et al., 2012; Sung et al., 2013; Zhang et al., 2014). Furthermore, inhibiting HDAC6 rescues the reduced mitochondrial axonal transport and mitochondrial length in hippocampal neurons treated with Aβ (Kim et al., 2012), as well as in pluripotent stem cells (iPSCs) from Amyotrophic Lateral Sclerosis (ALS) patients (Guo et al., 2017). In fact, due to its safety profile, HDAC6 is currently being considered as one of the most promising epigenetic targets in AD. Given the above, and despite the fact that CM-414 acts as a symptomatic and disease-modifying agent in AD mice models (Cuadrado-Tejedor et al., 2017), it is possible that some toxicity may be associated with the inhibition of the class I HDAC1, precluding its use in the chronic treatment of AD patients. Thus, in order to improve the safety profile of CM-414, we synthesized a new compound, CM-695, with higher selectivity for HDAC6 over class I HDACs.

PDE9 is a cyclic guanosine monophosphate (cGMP) specific PDE and it is the PDE most strongly expressed in the brain (Andreeva et al., 2001). In fact, when we compared the expression of PDE5 and PDE9 in the mouse hippocampus, we found that PDE9 is expressed 10 times more strongly than PDE5 (Supplementary Figure S1). Interestingly, the expression of PDE5 and PDE9 were increased in the cortex of AD patients compared to age-matched control subjects. Accordingly, levels of cGMP were decreased in the cerebrospinal fluid (CSF) of AD patients compared to that of healthy control individuals (Ugarte et al., 2015). By restoring cGMP levels through PDE5 and 9 inhibition, intracellular signaling pathways that are important in memory and learning could be stimulated. For example, an activation of the cAMP-responsive element binding protein (CREB) transcription factor can be observed, a factor known to be crucial for synapse formation and memory consolidation (García-Osta et al., 2012; Heckman et al., 2017). Since PDE9 has the highest affinity for cGMP of all the PDEs (Singh and Patra, 2014), it becomes an attractive target to increase the GMP in the brain. It was recently proposed that PDE9 inhibitors provide more protection against Aβ42 than PDE4 and PDE5 inhibitors in an *in vitro* model of AD (Cameron et al., 2017). Nevertheless, when specific PDE9 inhibitors (PF-04447943 and BI-409306) have been tested to treat AD in Phase II clinical trials, they both failed to meet their AD efficacy endpoints relative to the placebo (Schwam et al., 2014; Frölich et al., 2019). As indicated above, the complexity of the AD pathology means it is possible that the inhibition of a single enzyme alone will not produce therapeutic benefits in patients. Accordingly, we designed a new first-in class dual activity compound CM-695, that targets HDAC6 and PDE9 for inhibition, and with acceptable brain permeability, for it’s *in vivo* efficacy in Tg2576 mice.

## Materials and Methods

### Biological Activity *in vitro* and *in vivo*

Cells of the human neuroblastoma SH-SY5Y cell line were plated in 6-well plates and incubated at 37°C in a humid atmosphere with 5% CO_2_ until reaching a confluence of 80%–90%. They were cultivated in Eagle’s medium modified by Dulbecco (DMEM, Gibco BRL, Grand Island, NY, USA) supplemented with 1% non-essential amino acids solution (Gibco BRL, Grand Island, NY, USA), penicillin/streptomycin 100 U/ml (Gibco BRL, Grand Island, NY, USA) and 10% fetal bovine serum (Gibco BRL, Grand Island, NY, USA). Cells were incubated with different concentrations of CM-695 for 2 h. After incubation, medium was removed, washed with PBS and cells were lysed in a buffer containing SDS 2%, Tris-HCl (10 mM, pH 7.4), protease inhibitors (Complete Protease Inhibitor Cocktail, Roche) and phosphatase inhibitors (0.1 mM Na_3_VO_4_, 1 mM NaF). The homogenates were sonicated for 2 min and centrifuged at 14,000× *g* for 15 min.

Protein concentration was determined using the Pierce^TM^ BCA Protein Assay kit (Thermo Fisher Scientific, Waltham, MA, USA). For western blot analysis of acetylated histone 3 at lysine 9 (AcH3K9), pCREB and acetylated tubulin, protein samples (15–20 μg) were mixed with 6× Laemmli sample buffer and resolved onto SDS-polyacrylamide gels and transferred to nitrocellulose membrane. Membranes were blocked for 1 h with 5% milk in TBS and incubated overnight with the corresponding primary antibody: rabbit monoclonal anti-AcH3K9, rabbit monoclonal anti-pCREB (Ser129; 1:1,000, Cell Signaling Technology, Danvers, MA, USA), mouse monoclonal anti-Acetylated tubulin, mouse monoclonal anti-β-actin (1:50,000, Sigma-Aldrich, St. Luis, MO, USA).

### Animals and Treatments

Transgenic mice (Tg2576) between 16 and 18 months of age and female gender were used. This strain expresses the human 695-aa isoform of the amyloid precursor protein (APP) carrying the Swedish (K670N/M671L) familial AD mutation driven by a hamster prion promoter (Hsiao et al., 1996). Mice were on an inbred C57BL/6/SJL genetic background. The Tg2576 AD mice strain accumulates Aβ peptide exponentially, in the brain, between 7 and 12 months of age, showing a hippocampal damage from the age of 9–10 months (Chapman et al., 1999; Westerman et al., 2002).

Animals were bred at “Centro de Investigación Médica Aplicada” (CIMA) in Pamplona, Spain. Animals were housed 4–5 per cage with free access to food and water and maintained in a temperature controlled environment on a 12 h light-dark cycle. All procedures were carried out in agreement with the European and Spanish regulations (2010/63/EU; RD1201/2005), and the study was approved by the Ethical Committee for the Animal Experimentation of the University of Navarra.

Tg2576 mice were treated six times a week for 4 weeks. They were administered intraperitoneally with CM-695 (40 mg/kg *n* = 10) or vehicle (10% DMSO, 10% Tween-20 in saline solution). The preparation of drug was performed daily to avoid precipitation due to their hydrophobic nature. Behavioral and biochemical studies were performed comparing transgenic mice to age-and strain-matched transgenic negative littermates (WT). The behavioral tests were always carried out during light time (from 9 am to 14 pm), in order to minimize the possible influence of circadian rhythms.

### Behavioral Studies

Behavioral studies were carried out during light time (from 9 am to 2 pm). Protocols were approved by the Ethical Committee of the University of Navarra (in accordance with the European and Spanish Royal Decree 1201/2005).

#### Fear Conditioning Test

To evaluate the effects of drugs on cognitive function a fear-conditioning (FC) paradigm was used after 2 weeks of treatment with CM-695 (*n* = 11) or vehicle (*n* = 10). The FC is an hippocampus-dependent test to measure long-term memory consolidation by assessing the association between two stimuli, one conditioned (context) and another unconditioned (an electric shock; Maren, 2008). The conditioning procedure was carried out in a StartFear system (Panlab S.L., Barcelona, Spain) as described previously with slight modifications (Ricobaraza et al., 2012). During training phase mice received two footshocks (0.3 mA, 2 s) separated by an interval of 30 s. After 24 h mice were returned to the conditioning chamber and freezing behavior was recorded during 2 min.

#### Morris Water Maze Test (MWM)

During the 3rd week of treatment, Tg2576 mice treated with CM-695 (*n* = 11) or vehicle (*n* = 10) and non-transgenic littermates (*n* = 10) underwent spatial reference learning and memory testing in the Morris water maze (MWM) test (Morris, 1984) as previously described (Westerman et al., 2002). In this case, the hidden-platform training (with all visible cues present) was conducted during nine consecutive days (four trials per day) and memory retention was analyzed with three probe trials at the beginning of days 4th, 7th and 9th. Four animals per groups were sacrificed for hippocampal gene expression analysis 24 h after the last probe trial and the remaining animals were maintained to perform a reversal phase of MWM after a washout period of 4-weeks. In this phase, the platform was placed in the opposite quadrant of the tank and the hidden platform training during five consecutive days (four trials per day) was performed. All cues remained in their original positions. Memory retention was analyzed in a probe at day 6. Mice were monitored by a camera mounted in the ceiling directly above the pool, and all trials were recorded using an HVS water maze program for subsequent analysis of escape latencies and percent time spent in each quadrant of the pool during probe trials (analysis program WaterMaze3, Actimetrics, Evanston, IL, USA). All experimental procedures were performed blind to groups. Animals were euthanized 24 h after the last probe.

### Determination of Aβ Levels

Aβ_42_ pool containing intracellular and membrane-associated Aβ_42_ levels were measured in the parieto-temporal cortical extracts by using a sensitive sandwich ELISA kit (Invitrogen, Camarillo, CA, USA). Tissue was homogenized in a buffer containing SDS 2%, Tris-HCl (10 mM, pH 7.4), protease inhibitors (Complete Protease Inhibitor Cocktail, Roche) and phosphatase inhibitors (0.1 mM Na_3_VO_4_, 1 mM NaF). The homogenates were sonicated for 2 min and centrifuged at 100,000× *g* for 1 h. Aliquots of supernatant were directly diluted and loaded onto ELISA plates in duplicate. The assays were performed according to the manufacturer’s instructions.

### Affymetrix Microarray Hybridization and Data Analysis

The hippocampi were dissected and RNA was extracted with TRIzol Reagent (Invitrogen, Carlsbad, CA, USA) and purified with the RNeasy Mini-kit (Qiagen, Hilden, Germany). RNA integrity was confirmed on Agilent RNA Nano LabChips (Agilent Technologies, Santa Clara, CA, USA). The sense cDNA was prepared from 300 ng of total RNA using the Ambion^®^ WT Expression Kit. The sense strand cDNA was then fragmented and biotinylated with the Affymetrix GeneChip^®^ WT Terminal Labeling Kit (PN 900671). Labeled sense cDNA was hybridized to the Affymetrix Mouse Gene 2.0 ST microarray according to the manufacturer protocols and using GeneChip^®^ Hybridization, Wash and Stain Kit. Genechips were scanned with the Affymetrix GeneChip^®^ Scanner 3,000. Microarray data files were submitted to the GEO (Gene Expression Omnibus) database and are available under accession number GSE128422.

Both background correction and normalization were done using RMA (Robust Multichip Average) algorithm (Irizarry et al., 2003). Then, a filtering process was performed to eliminate low expression probe sets. Applying the criterion of an expression value greater than 16 in at least three samples for each experimental condition (hippocampi from mice treated with vehicle or CM-695, with three biological replicates for condition), 22,191 probe sets were selected. R/Bioconductor (Gentleman) was used for preprocessing and statistical analysis.

First, we applied one of the most widely used methods to find out the probe sets that showed significant differential expression between experimental conditions, LIMMA (Linear Models for Microarray Data; Wettenhall and Smyth, 2004). Genes were selected as significant using *p*-value < 0.01 as threshold. Using False Discovery Rate (FDR) method to correct for multiple hypotheses testing no significant results was obtained.

Additional network and functional analyses were analyzed through the use of IPA (QIAGEN Inc.^1^).

### Quantitative Real-Time PCR

The RNA was treated with DNase at 37°C for 30 min and reverse-transcribed into cDNA using SuperScript^®^ III Reverse Transcriptase (Invitrogen, Carlsbad, CA, USA). Quantitative real-time PCR (QRT-PCR) was performed to quantified gene expression. All the assays were done in triplicate using Power SYBR Green PCR Master Mix (Applied Biosystems, Warrington, UK) and the corresponding specific primers for heat shock protein family A (Hsp70) member 1 A/B (HSPA1A/B; Fw: 5′-AGCCTTCCAGAAGCAGAGC-3′; Rev: 5′-GGTCGTTGGCGATGATCT-3′), 78-KDa glucose regulated protein (GRP78; Fw: 5′-ACCAACTGCTGAATCTTTGGAAT-3′; Rev: 5′-GAGCTGTGCAGAAACTCCGGCG-3′) and for the internal control 36B4 (5′-AACATCTCCCCCTTCTCCTT-3′; 5′-GAAGGCCTTGACCTTTTCAG-3′). Real-time was carried out using an ABI Prism 7300 sequence detector (Applied Biosystems, Foster City, CA, USA) and data were analyzed using the Sequence Detection software v.3.0 (Applied Biosystems, Foster City, CA, USA). The relative gene expression was calculated with reference to the control group using the DDCT method (Livak and Schmittgen, 2001).

### Dendritic Spine Density Measurement by Golgi-Cox Staining

A modified Golgi-Cox method (Glaser and Van der Loos, 1981) was used to analyze dendritic spine density. Half-brains were incubated in Golgi-Cox solution (1% potassium dichromate, 1% mercury chloride, 0.8% potassium chromate) for 48 h at RT (protected from light). The solution was then renewed and tissue was maintained there for another 3 weeks. Thereafter, brains were maintained in 90° ethanol for 30 min until being processed in 200 μm-thick coronal slices using a vibratome. The slices were incubated in 70° ethanol, reduced in 16% ammonia for 1 h and fixed in 1% sodium thiosulfate for 7 min. They were then dehydrated in an increasing alcohol graduation and mounted with DPX mountant (VWR International, Leuven, Belgium).

Spine density was determined in the secondary apical dendrites of the pyramidal cells located within the CA1 region of the hippocampus (Megías et al., 2001). Each selected neuron was captured using a Nikon Eclipse E600 light microscope and images were recorded with a digital camera (Nikon DXM 1200F) at a resolution of 1,000–1,500 dots per inch (dpi). For each mouse (*n* = 3), three dendrites of nine different neurons were used for the analysis.

### Data Analysis and Statistical Procedures

The data were analyzed with SPSS for Windows, version 15.0 (SPSS, Chicago, IL, USA) and unless otherwise indicated, the data are expressed as means ± standard error of the mean (SEM). Normal distribution of data was checked by the Shapiro–Wilks test.

In the MWM, latencies to find the platform were examined by two-way repeated measures analysis of variance (ANOVA) test (genotype × trial) to compare the cognitive status in WT mice and Tg2576 mice. Likewise, the treatments effect in spatial memory was examined also by a two-way repeated measures ANOVA test (treatment × trial) followed by *post hoc* Scheffe’s analysis. When two groups were compared, Student’s *t*-test was used, whereas when more than two experimental groups were compared, one-way ANOVA followed by *post hoc* Scheffe’s test was used.

## Results

### Biological Activity

**Figure 1:**
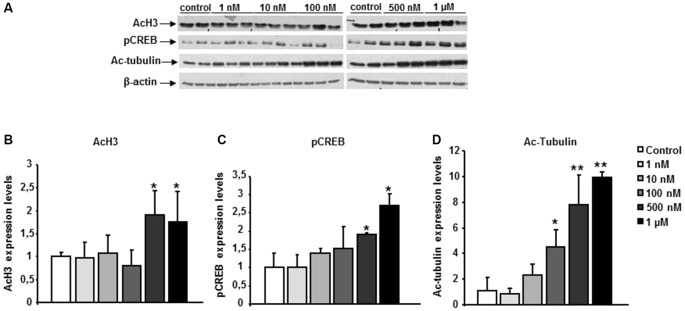
CM-695 shows *in vitro* functional activities. **(A)** Representative bands of western blots showing histone 3 acetylation at lys 9 (AcH3K9), pCREB and Ac-Tubulin levels in SH-SY5Y cells treated with CM-695 at different concentrations (1 nM to 1 μM) for 2 h. **(B–D)** Histograms show the quantification of the immunochemically reactive bands in the western blot, *n* = 3 wells per condition, repeated in three different cultures. β-actin was used as a loading control. Data are represented as mean ± standard error of the mean (SEM) expressed as the fold change vs. vehicle; *p < 0.05, **p < 0.01.

The functional activity of the chemical probe CM-695 against its targets (HDAC class I, PDE9 and HDAC6) was assessed *in vitro*, in SH-SY5Y neuroblastoma cells. These cells were exposed to CM-695 for 2 h at concentrations ranging from 1 nM to 1 μM in order to determine its effect on histone acetylation (lysine 9 of histone 3, H3K9 mark), CREB-Ser133 phosphorylation and α-tubulin acetylation by western blot analysis. There was a significant increase in α-tubulin acetylation (4.47 ± 1.36 fold change vs. control, *p < 0.05, Figure 1D) but not of AcH3K9 (0.81 ± 0.33 fold change vs. control, Figure 1B) in SH-SY5Y cells exposed to CM-695 (100 nM), consistent with its selective inhibition of HDAC6 (IC50 = 40 nM) as opposed to class I HDACs (IC50 = 593 and 3530 nM for HDAC1 and HDAC2, respectively). However, CM-695 had a significant effect on AcH3K9 at 500 nM (1.91 ± 0.56 fold change vs. control, *p < 0.05, Figures 1A,B), although this effect was much stronger on tubulin acetylation (7.78 ± 2.36 fold change vs. control, **p < 0.01, Figures 1A,D). In addition, at a concentration of 500 nM CM-695 significantly increased the levels of pCREB (1.91 ± 0.02 fold change vs, control, *p < 0.05, Figures 1A,C), indicating this compound acts also against PDE9 (IC50 = 107 nM). These data confirmed the functional activity of this compound *in vitro* against its targets (HDAC6, PDE9 and HDAC class I).

### CM-695 Ameliorates Memory Impairment in Aged Tg2576 Mice

Pharmacokinetic studies described for this compound indicated that CM-695 reached an acceptable brain concentration (around 100 nM/kg) 15 min after administering a dose of 40 mg/kg, concentrations that, according to the IC50, would ensure an effect on HDAC6 and PDE9. Furthermore, functional response in mouse brain was assessed *in vivo* in a group of animals to demonstrate the ability of the compound to inhibit HDAC and PDE9. Fifteen, 30 and 60 min after i.p. injection of 40 mg/Kg mice were sacrificed by cervical dislocation and their hippocampus were dissected. A western-blot was carried out to analyze pCREB and Ac-Tubulin in the hippocampus. As it shown in the Supplementary Figure S2, CM-695 increased pCREB levels at 15, 30 and 60 min. Regarding Ac-Tubulin, as basal levels of this protein are high in the brain of the animals, levels were slightly increased but no significant differences were appreciated. Likewise, a higher effect would probably be obtained with longer exposing times or in a chronic treatment. These data, demonstrated that CM-695 cross the BBB and reach the brain at a concentration which is enough to inhibit PDE9 (IC50 107 nm). Considering that HDAC6 IC50 is 40 nM and that a chronic treatment is achieved, an effect on Ac-Tubulin was assumed.

**Figure 2:**
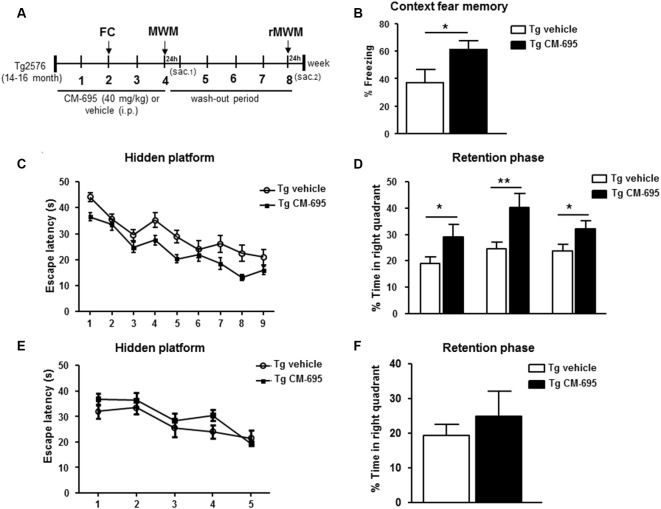
Chronic treatment with CM-695 reversed learning deficits in aged Tg2576 mice. **(A)** Scheme showing timeline for treatment, behavioral tasks, and killing of mice. FC, fear conditioning; MWM, Morris water maze; rMWM, reversal MWM; Sac, sacrificed. **(B)** Freezing behavior from Tg2576 mice treated with vehicle (*n* = 10) or CM-695 (*n* = 11). Data represent the percentage of time of freezing during a 2 min test. **(C)** Escape latency of the hidden platform in the MWM test for the Tg2576 mice treated with vehicle (*n* = 10) or CM-695 (*n* = 11). **(D)** Percentage of time spent in correct quadrant during the probe test (days 4, 7, and 9). **(E)** Escape latency during the rMWM test for the Tg2576 mice treated with vehicle (*n* = 10) or CM-695 (*n* = 11) after the washout period. **(F)** Percentage of time spent in correct quadrant during the probe test after rMWM phase (day 6). In all figures results are expressed as mean ± SEM; *p < 0.05, **p < 0.01.

We analyzed the effects of administering CM-695 on the memory impairment in aged Tg2576 mice after a 2-week treatment (40 mg/kg, i.p. daily), assessing their performance in the fear conditioning test, a hippocampus-dependent learning task (Supplementary Figure S3A). Interestingly, the freezing response of Tg2576 animals was significantly (*p < 0.05) stronger in those mice that received CM-695 (60.9% of freezing) than in those that received the vehicle alone (36.9% of freezing, Figure 2A). Moreover, 1 week later we evaluated the effects of CM-695 administration on learning in the MWM test (Supplementary Figure S3B). While the latency to find the platform was prolonged in Tg2576 mice relative to the WT mice, the escape latency was shorter when these Tg2576 mice received CM-695, although globally no significant differences were found between groups (Figure 2B). Interestingly, the Tg2576 mice remained significantly longer time in the target quadrant during the probe tests on days 4th (29.08 ± 4.6% vs. 18.91 ± 2.52%, *p < 0.05), 7th (40.13 ± 5.50% vs. 24.61 ± 2.62%, **p < 0.01) and 9th (32.18 ± 3.0% vs. 23.70 ± 2.45%, *p < 0.05) when they received CM-695 (Figure 2C). To determine whether the effect of CM-695 persisted when the mice no longer received this compound, mice were re-trained in a reversal phase of the MWM test after a month wash-out, placing the platform in the opposite quadrant. The hidden platform training was carried out over 5 days (four trials per day) and it was followed by a memory retention test on day 6. No significant differences were found between the Tg2576 mice that received CM-695 or the vehicle alone in the hidden platform phase (Figure 2D) or in the probe trial (Figure 2E), indicating that none of the mice learned the platform location. Thus, it appears that the effect of CM-695 did not persist after the 4-week wash-out period.

Taking all these results into account, it can be inferred that a chronic treatment with CM-695 ameliorated memory impairment in aged-Tg2576 mice although its effect was lost after a wash-out period of 4 weeks.

### CM-695 Diminishes the Pathological Markers of AD in Aged-Tg2576 Mice

**Figure 3:**
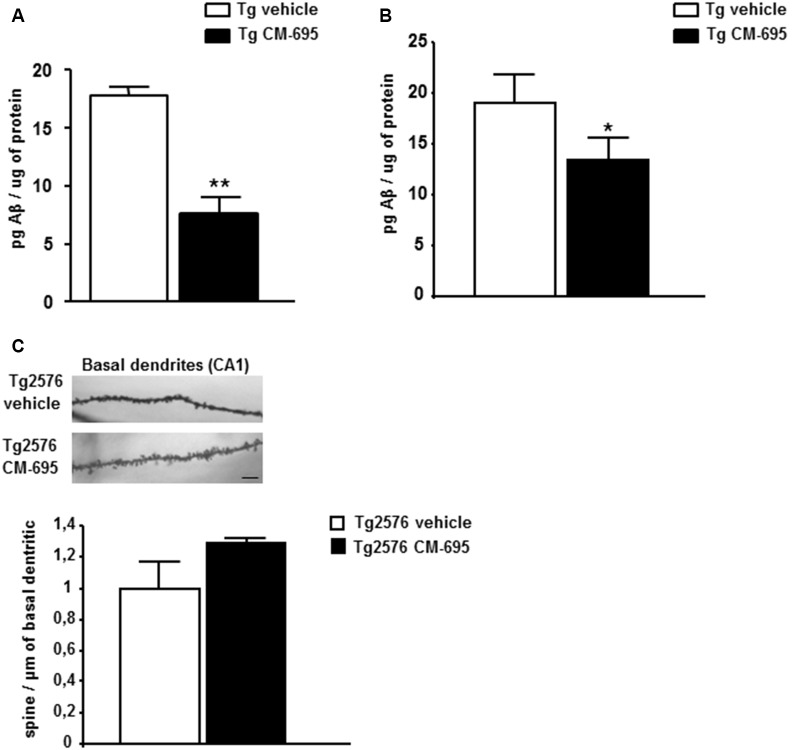
Chronic treatment with CM-695 decreased amyloid levels but did not affect hippocampal dendritic spine density. Aβ_42_ levels determined by ELISA in SDS hippocampal tissue extracts of Tg2576 mice treated with vehicle or CM-695 after 4 week of treatment (*n* = 4; **A**) or after a washout period of 4 weeks (*n* = 6–7). **(B)** *p < 0.05, **p < 0.01. **(C)** Representative Golgi staining images of the apical dendrites on CA1 hippocampal pyramidal neurons. Scale bar, 10 μm. The histograms represent the quantification of spine density of basal dendrites of hippocampal CA1 pyramidal neurons from Tg2576 mice treated with vehicle or CM-695 (*n* = 34–36 neurons from three animals per group).

To determine the effects of CM-695 on amyloid pathology in aged-Tg2576 mice, ELISA was used to assess the Aβ_42_ in parieto-temporal cortical extracts (see “Materials and Methods” section). The Aβ_42_ levels were measured in two different groups of animals: one sacrificed at the end of the first MWM (after the 4th week of treatment, *n* = 4 per condition) and the other sacrificed at the end of the reversal-MWM (after the 4-week wash-out period in which the mice were not treated, *n* = 6–7 per condition). There was a significant decrease in Aβ_42_ in the Tg2576 mice treated with CM-695 and sacrificed at the end of the treatment (7.70 ± 2.07, **p < 0.01) relative to those that received the vehicle alone 17.8 ± 1.40, Figure 3A). Moreover, no Aβ_42_ was detected in the WT littermates (data not shown). However, it was noteworthy that there was a significant decrease in the hippocampal Aβ_42_ in the group of animals that was sacrificed after the 4-week wash-out period (13.33 ± 2.1, vs. 19.05 ± 2.69, *p < 0.05, Figure 3B), although these differences (45% reduction) were not as strong as those observed prior to the wash-out period (56% reduction). These results suggest that a chronic treatment with CM-695 decreased amyloid pathology in elderly Tg2576 mice.

Given that Tg2576 mice display synaptic loss and dysfunction (Ricobaraza et al., 2012), we assessed whether the behavioral recovery induced by CM-695 was reflected by structural changes in dendritic spine density. Consistent with previous data, there was a significantly lower density of apical dendrites on CA1 pyramidal neurons in Tg2576 mice than in WT mice (Supplementary Figure S4). Taking into account the behavioral data obtained at the end of the treatment (Figure 2), we assumed that they could correlate with an increase in the density of dendritic spines in the treated animals, thus, we analyzed if the effect was maintained after 4 weeks without treatment as we observed for CM-414. However, when the mice were analyzed after the wash-out period, those that received CM-695 did not show any significant change in the density of spines on these neurons. It should be noted that there was a tendency to increase the density of spines that might account for the memory improvement observed 4-weeks after treatment (Figure 3C). Nevertheless, after the wash-out period neither the effect on memory function nor the potential changes in dendritic spine density persisted.

**Figure 4:**
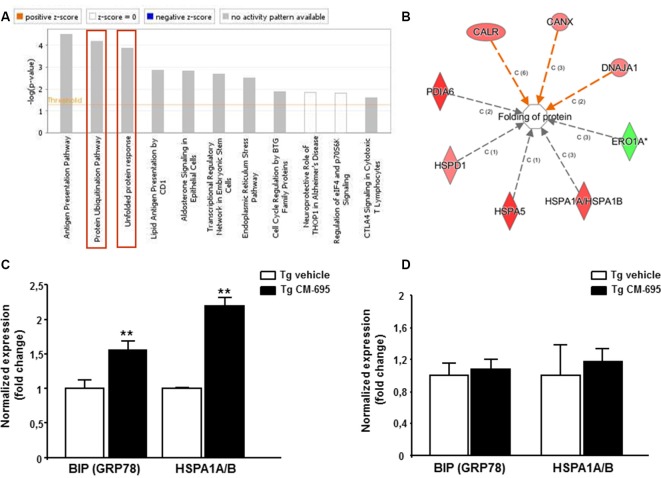
Chronic treatment with CM-695 significantly increases chaperones: GRP78 and HSP70. **(A)** Ingenuity pathway analysis showing the most highly scoring canonical pathways (according to *p*-value). Horizontal orange line running through the bars is the threshold for *p*-value for these pathways. Color coding for positive and negative *z*-score and for pathways with no activity pattern available are shown in the figure. “Protein Ubiquitination Pathway” and the “Unfolded protein response” (remarked with a red box) were included among the top-ranked canonical pathways. **(B)** Network of differentially expressed genes categorized in Ingenuity by Disease and biological functions. **(C,D)** Quantitative RT-PCR (QRT-PCR) analysis of BIP (GRP78) and HSPA1A/B mRNA in the hippocampus of CM-695 treated mice vs. vehicle, 4 weeks after treatment (**C**, *n* = 4) and after the washout period respectively (**D**, *n* = 6–7). Bars represent the fold change (mean ± SEM) in gene expression normalized to vehicle-treated mice; **p < 0.01.

To explore the mechanisms underlying the effect of CM-695 on amyloid pathology and on memory function, we analyzed the effects of CM-695 on gene expression in the hippocampus of Tg2576 mice compared to a group of transgenic animals receiving vehicle using Affymetrix microarrays. LIMMA was applied to find out the probe sets that showed significant differential expression between experimental conditions (Smyth, 2004). Genes were selected as significant using *p* < 0.01 as threshold. Differentially expressed genes were analyzed by using Ingenuity Pathways Analysis in order to gain information about the mechanistic approach of CM-695 therapeutic effect. Importantly, the Protein Ubiquitination Pathway (*p*-value = 6.76, E-05) and the Unfolded protein response (UPR, *p*-value = 3.1, E-05) were included among the top-ranked canonical pathways (Figure 4A) and more specifically, BIP (GRP78) and HSPA1A/B were among the genes overexpressed in the hippocampus of mice administered with CM-695 respect to the mice receiving vehicle (Supplementary Figure S5). In accordance, when differentially expressed genes were also categorized to diseases and biological functions “folding protein” (*p*-value and molecules) was significantly regulated (Figure 4B). Since chaperone activity plays a crucial role in proper protein folding activity, we analyzed the expression of GRP78 and HSPA1A/B by quantitative real time PCR. Accordingly to the results obtained in the array, a significant (*p* < 0.01) increase was observed in both GRP78 and HSPA1A/B mRNA levels in the hippocampus of CM-695 treated mice respect to mice receiving vehicle (Figure 4C). Next, we checked if this effect in gene expression was maintained after the washout period. As depicted in Figure 4D, animals receiving CM-695 and sacrificed after a 4-weeks wash out period showed similar expression levels of GRP78 and HSPA1A/B to control group.

These results suggest that the increase in the levels of chaperones GRP78 and Hsp70, which are involved in protein folding, may underlie the improvement in AD symptoms observed after daily administration of the compound.

## Discussion

Given the high failure rate in AD drug development, with no new drug having been approved for this diseases since 2003 (Cummings et al., 2014), it is clearly necessary to change the way we approach the search for new AD targets to improve the results obtained in clinical trials. We have identified a NCE, CM-695, that has potential therapeutic effects in a well-established mouse model of AD. CM-695 is a potent HDAC6 and PDE9 inhibitor that, after chronic administration, ameliorates the cognitive impairment and amyloid pathology evident in aged-Tg2576 mice. An increase in the expression of the chaperones GRP78 and Hsp70, involved in protein folding, may underlie the improvement in AD symptoms observed after daily administration of the compound. The benefits obtained with this dual inhibitor are not maintained when the compound is no longer administered. Thus, CM-695 appears to be a safe and efficient disease-modifying agent for AD treatment, confirming that multi-target therapies may represent better options for the treatment of complex diseases.

HDACs are emerging targets for the treatment of AD (Yang et al., 2017). The inhibition of HDAC class I facilitates gene transcription and the formation of new synapses (Rumbaugh et al., 2015). The inhibition of HDAC class IIb (HDAC6), by targeting cytoplasmic proteins is involved in the clearance of misfolding proteins (Sung et al., 2013). The inhibition of both isoforms could be a promising and synergistic therapy to treat AD, which has been demonstrated by using pan- HDAC inhibitors. However, these compounds have unfavorable side effects when administered chronologically and at a dose sufficient to reach the brain at appropriate concentrations. One strategy to reduce toxicity while maintaining effectiveness is to obtain new disease-modifying molecules maintaining HDAC inhibition combined with an additional function such as inhibition of PDEs, inhibition of glycogen synthase kinase 3β or antioxidant activity (De Simone and Milelli, 2019).

Based on the results obtained previously with different selectivity profiles of HDAC and PDE inhibitors (Rabal et al., 2016, 2018, 2019; Cuadrado-Tejedor et al., 2017; Sánchez-Arias et al., 2017), we designed a new series of PDE9 inhibitors that more specifically target HDAC6 over class I HDAC isoforms. This shift in specificity aimed to reduce the toxicity associated with class I inhibition which complicates the further therapeutic development of the new compound. Furthermore, since PDE inhibitors have pro-cognitive and neuroprotective effects, the inhibition of PDE9 was selected rather than PDE5 as its brain expression and affinity for cGMP is higher than that of other PDEs (Supplementary Figure S1, Andreeva et al., 2001; Singh and Patra, 2014). Among the new compounds designed, CM-695 was tested in AD model following the same guidelines used with CM-414 (Cuadrado-Tejedor et al., 2017), in order to compare the efficacy between the two compounds. The therapeutic effects obtained after chronic treatment with CM-695 were similar to those obtained with CM-414, except that the effect did not persist when the drug was no longer administered. Taking into account the differences in the inhibition profile of the two compounds, we confirm that the inhibition of class I HDACs, and more specifically HDAC2, plays an important role in maintaining memory function.

Interestingly, the effect of CM-695 on Aβ clearance is maintained, which may be mediated by the inhibition of HDAC6 (Boyault et al., 2007). One of the targets of HDAC6 is heat-shock protein 90 (Hsp90). As such, the inhibition of HDAC6 increases Hsp90 acetylation, releasing its client proteins like heat shock transcription factor 1 (HSF1), which in turn translocates to the nucleus and mediates the transcription of HSPA1A/B genes, and ultimately, Hsp70 (Wang et al., 2014). HSPA1A/B was among the genes overexpressed in the Affymetrix microarray analysis and validated by qRT-PCR in the hippocampus of mice administered CM-695. Significantly, a similar induction was induced by CM-695 in a model of thrombosis (Allende et al., 2017). Hsp70 fulfills a neuroprotective role in AD by decreasing the oligomerization and production of toxic Aβ isoforms, and by increasing its degradation (Magrané et al., 2004; Muchowski and Wacker, 2005; Kumar et al., 2007). Interestingly, Hsp70 upregulation by inhibiting Hsp90 was also proposed as a mechanism to normalize synaptic transmission in a transgenic model of tau aggregation (Thirstrup et al., 2016). These results suggest that the increase in Hsp70 expression observed at the end of treatment is at least partially responsible for the marked decrease in hippocampal Aβ levels observed. Together with PDE9 inhibition, this increase in Hsp70 may help restore learning ability. However, Hsp70 was no longer upregulated 4 weeks after the end of the treatment, causing a loss in Aβ clearance and a deterioration of the animals learning capacity. Considering the safe profile of CM-695, chronic treatment with no need for wash-out periods could be an option to consider for this NCE.

In conjunction, it seems that CM-695 is a safe and efficient disease-modifying agent to treat AD, confirming that multitarget therapies may provide good options to combat AD, as seen in other multifactorial diseases like cancer or AIDS.

## Data Availability

The raw data supporting the conclusions of this manuscript will be made available by the authors, without undue reservation, to any qualified researcher.

## Ethics Statement

All procedures were carried out in agreement with the European and Spanish regulations (2010/63/EU; RD1201/2005), and the study was approved by the Ethical Committee for the Animal Experimentation of the University of Navarra.

## Author Contributions

AG-O, MC-T and JO conceived the general framework of this study, designed experiments and discuss results. MP-G, CG-M, DM and CG-B performed the *in vitro* functional assays in cell culture, treatments, behavioral experiments and biochemical assays. JS-A performed biochemical experiments related to CM-695 compound *in vitro*. VS performed the bioinformatic analysis of the microarrays. OR and JO designed and characterized CM-695. AG-O and MC-T wrote the manuscript.

## Conflict of Interest Statement

The authors declare that the research was conducted in the absence of any commercial or financial relationships that could be construed as a potential conflict of interest.

## Footnotes

^1^https://www.qiagenbioinformatics.com/products/ingenuity-pathway-analysis/
