# Supplementary material for: Taking Advantage of the Selectivity of Histone Deacetylases and Phosphodiesterase Inhibitors to Design Better Therapeutic Strategies to Treat Alzheimer’s Disease
Source: Front Aging Neurosci. 2019 Jun 21;11:149. doi: 10.3389/fnagi.2019.00149 (PMC6597953; doi:10.3389/fnagi.2019.00149)
Supplement: Supplementary file 1 [file Data_Sheet_1.docx]

Supplementary Material

# Supplementary Figures and Tables

## Supplementary Figures


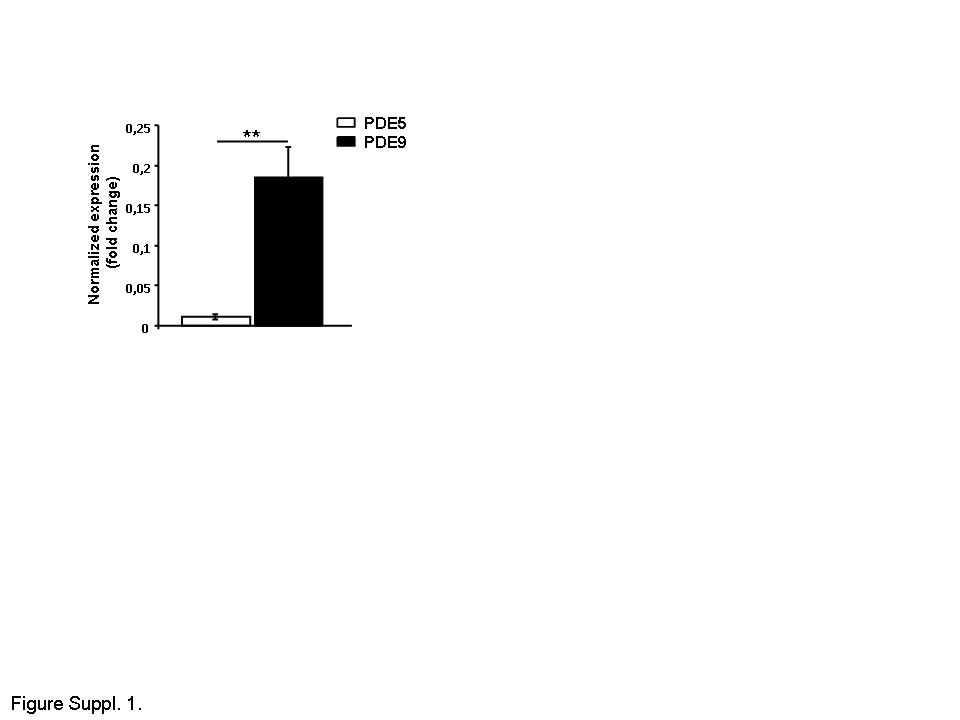


**Supplementary Figure S1. Significant higher PDE9 vs PDE5 mRNA expression levels in mice hippocampi.** Mouse hippocampal PDE5 and PDE9 mRNA levels were analyzed by quantitative RT-PCR ***p*<0.01.


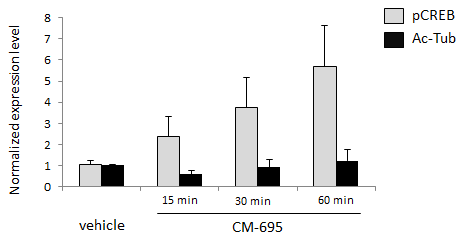


**Supplementary Figure S2.** Quantification of western blots showing pCREB and Ac-Tubulin (Ac-Tub) levels in hippocampal extracts from mice sacrificed 15, 30 min and 60 min after treatment with CM-695 (40 mg/kg). Actin was used as a loading control (n = 3 per group).


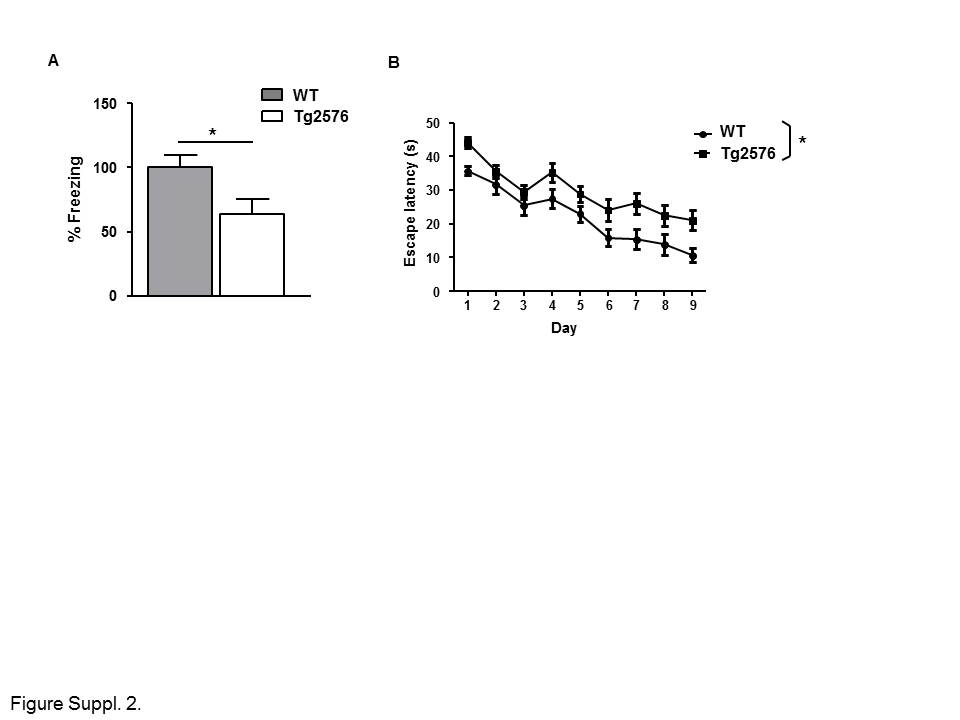
**Supplementary Figure S3. Tg2576 mice show memory deficits in the FC and MWM test. (A)** Freezing behavior from 14-16 month old Tg2576 compared to age-matched WT mice (**p* ≤ 0.05). Data represent the percentage of time freezing during a 2-min test. **(B)** Escape latency of the hidden platform in the MWM test for 14-16 month old-Tg2576 and WT mice (**p* ≤ 0.05). Results are expressed as mean ± SEM (n = 10–12 per group).


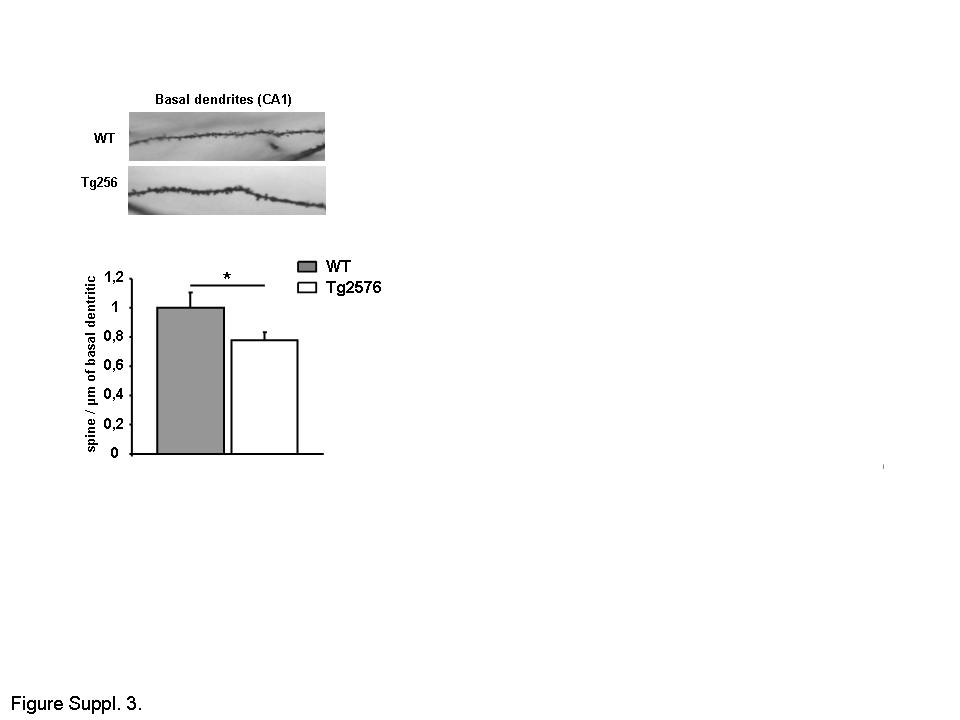


**Supplementary Figure S4. Tg2576 mice show significant reduction in spine density of basal hippocampal pyramidal neurons compared to aged-matched wild type mice (WT).** The histogram represents the quantification spine density of basal hippocampal pyramidal neurons from 14-16 month old Tg2576 compared to age-matched WT mice. Representative images of Golgi staining from CA1 dendrites are shown **p* < 0.05.


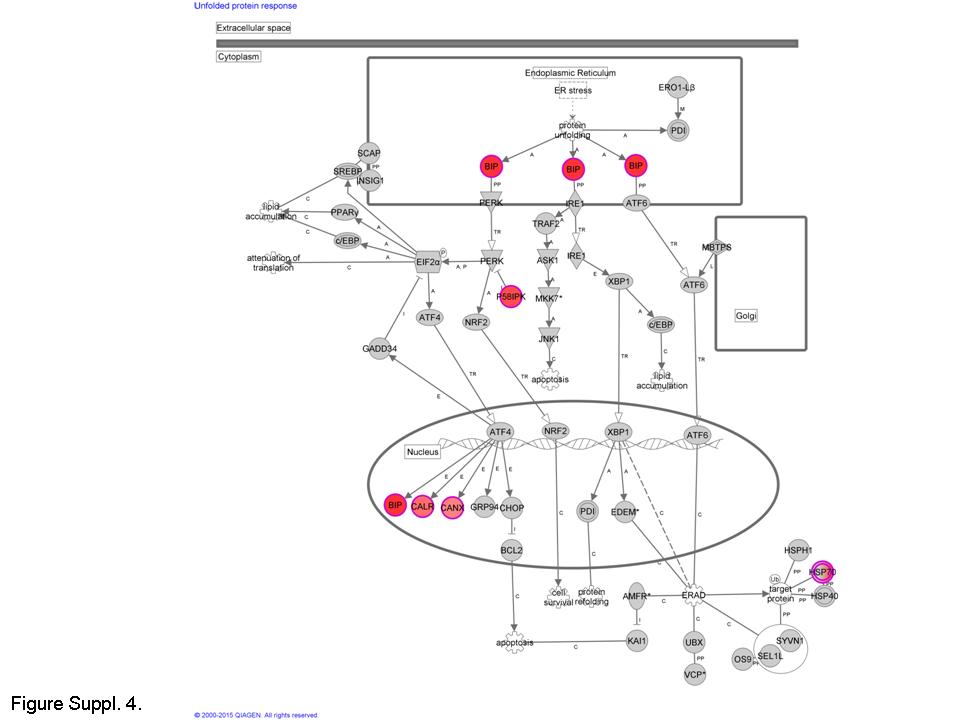


**Supplementary Figure S5. Upregulation of the unfolded protein response in the hippocampus of CM-695 treated mice.** Ingenuity schematic draw showing all regulated potential molecular players of the unfolded protein response. The filled (red) symbols represent the entries which are actually statistically significantly up-regulated by CM-695.
